# High Intestinal and Systemic Levels of Interleukin-23/T-Helper 17 Pathway in Chinese Patients with Inflammatory Bowel Disease

**DOI:** 10.1155/2013/425915

**Published:** 2013-12-05

**Authors:** Lu Song, Rui Zhou, Sha Huang, Feng Zhou, Shufang Xu, Wei Wang, Fengming Yi, Xiaobing Wang, Bing Xia

**Affiliations:** ^1^Department of Gastroenterology/Hepatology, Zhongnan Hospital of Wuhan University, Wuhan, Hubei 430071, China; ^2^Clinical Center & Key Laboratory of Intestinal & Colorectal Diseases of Hubei Province and Key Laboratory of Allergy and Immune-Related Disease, Wuhan, Hubei 430071, China

## Abstract

Interleukin-23/T-helper 17 (IL-23/Th17) pathway plays a key role in the pathogenesis of inflammatory bowel disease (IBD), but little is known about its expression in Chinese population. In this study, we investigated the mRNA and protein levels of IL-12p40, tumor necrosis factor-like cytokine 1A (TL1A), Janus kinase 2 (JAK2), and IL-23R both locally and systemically in Chinese IBD patients. Our results indicated that the mRNA levels of IL-12p40 and TL1A were increased in ulcerative colitis (UC) patients. Furthermore, serum IL-12p40 and TL1A levels were higher in active UC patients, especially in patients with disease course less than 1.25 years or initial onset. No correlation was found between the genotype and serum levels of IL-12p40 or TL1A in UC patients. Additionally, the mRNA and protein expression of JAK2 and IL-23R were increased in UC and Crohn's disease (CD) patients. Taken together, our results provided evidence that IL-23/Th17 pathway genes may represent important biomarkers of active stage of IBD and serve as novel therapeutic targets for IBD in Chinese population.

## 1. Introduction

Inflammatory bowel disease (IBD) is a chronic, relapsing inflammatory disorder of the gastrointestinal tract which includes ulcerative colitis (UC) and Crohn's disease (CD). IBD is caused by complex interactions of genetic, immunoregulatory factors, intestinal microbiota, and environmental factors. Of these, genetic susceptibility of IBD has been demonstrated as a key factor by traditionally epidemiological studies [1]. Genome-wide association (GWA) studies have discovered some IBD susceptibility genes in interleukin-23/T-helper 17 (IL-23/Th17) pathway, such as IL-12B, IL-23R, Janus kinase 2 gene (JAK2), signal transducer and activator of transcription 3 (STAT3) and tumor necrosis factor (ligand) superfamily member 15 (TNFSF15) [2–5].

So far, little is known about the IL-23/Th17 pathway in Chinese IBD patients, and many studies illustrate that genetic mutations that predispose to IBD appear to vary between different geographical and racial groups [6, 7]. Thus, our previous study examined the distribution of 26 SNPs of UC and 18 SNPs of CD in the IL-23/Th17 pathway genes in Chinese IBD patients and found that the polymorphisms of IL-12B, IL23R, JAK2, and TNFSF15 are strongly associated with Chinese IBD patients. It is illustrated that the IL-23/Th17 pathway is a key regulator of intestinal homeostasis and proinflammatory response in defense of microbial infection [8–10]. IL-12B encodes the IL-12p40 subunit shared by IL-12 and IL-23 cytokine on the genetic level [11]. Functionally, the proinflammatory cytokines IL-12 and IL-23 play critical roles in bridging the innate and adaptive immune systems in IBD, while IL-23R may play more important role than IL-12/23p40 in the genetic susceptibility to IBD [8, 12, 13].

The interplay of IL-23 and IL-23 receptor complex activates the JAK2/STAT3 signaling pathway and ultimately leads to a variety of downstream immune responses. Recently, JAK2 is demonstrated to be associated with increased risk of UC and CD in a large study across the United Kingdom [14]. Meanwhile, the first GWA study provides evidence that the variation in TNFSF15 leads to both CD and UC in the European population [15]. Moreover, the cytokine, tumor necrosis factor-like cytokine 1A (TL1A), encoded by the TNFSF15, is involved in the IBD pathogenesis [16]. Accumulating evidence demonstrates that the IL-23R SNPs might lead to a variant in the 39-untranslated region of IL-23R mRNA and affect its response to anti-TNF therapy in UC [17, 18]. So this association between single nucleotide polymorphisms (SNPs) and IBD may be explained by the effects on the gene function and/or expression leading to dysregulation of intestinal inflammation.

Overall, our laboratory illustrated the polymorphism of IL-23/Th17 pathway genes, while the phenotypic functions and potential genotype-phenotype interactions are mainly unknown in Chinese IBD patients. Here we investigate the mRNA and protein expression of IL-12B, TNFSF15, JAK2, and IL23R both locally (intestinal mucosal) and systemically (peripheral blood) in Chinese IBD patients to provide a revealing insight into their roles in IBD pathogenesis.

## 2. Materials and Methods

### 2.1. Subjects

In this study, 118 patients with UC and 30 patients with CD were studied, which were previously genotyped IL-23/Th17 genes polymorphisms (Table 1). The diagnosis was based on conventional clinical, radiological, endoscopic, and histological criteria. The extent of colonic disease was determined by endoscopy and reported according to the Montreal classification [19]. Disease activity was assessed by the Truelove and Witts activity index in UC patients [20], while CD patients were determined by the Crohn's disease activity index (CDAI) [15]. Comparison was made with 93 healthy controls while individuals with infectious colitis, ischemic colitis, intestinal tuberculosis, and autoimmune diseases were excluded. This study was approved by the ethics committee of Zhongnan Hospital of Wuhan University.

### 2.2. Quantitative Real-Time PCR Analysis

For mRNA expression analysis, intestinal mucosal biopsies were obtained during colonoscopic investigation in 31 UC patients, 30 CD patients, and 28 healthy controls. Real-time PCRs were performed with the real-time PCR kit (Takara, Shiga, Japan) and amplified in the LightCycler instrument (BioRad, Hercules, CA, USA). The reaction conditions were initial denaturation at 95°C for 30 s, followed by 40 cycles of denaturing at 95°C for 5 sec and annealing at 54°C (IL-12B), 58°C (TL1A), 55°C (JAK2), or 56°C (IL-23R) for 40 sec. All samples were processed in triplicate. Relative units were calculated by the 2^−ΔΔCT^ method [21]. The primer sets used for PCR amplification were shown (Table 2).

### 2.3. Detection of Soluble IL-12p40 and TL1A by ELISA

Blood was collected from 118 UC patients and 93 healthy controls in serum separator tubes, and serum was separated, aliquoted, and stored at −80°C. Serum IL-12B (Mabtech, Stockholm, Sweden) and TL1A (Enzo, Farmingdale, NY, USA) levels of UC patients and healthy controls were measured by enzyme-linked immunosorbent assay (ELISA), according to the manufacturer's protocol.

### 2.4. Western Blot

Proteins were extracted from colon tissues which we used for quantitative PCR mentioned above. Protein samples were separated on 10% SDS-PAGE gel electrophoresis and transferred to a polyvinylidene fluoride membrane (Millipore, Billerica, MA, USA), and then immunoblotted with primary antibodies: human JAK2 (Cell Signaling Technology, Beverly, MA, USA), human IL-23R (Millipore, Billerica, MA, USA), and *β*-actin (Cell Signaling Technology, Beverly, MA, USA), respectively. The blots were developed by enhanced chemiluminescence (ECL) Western Blotting Substrate (Pierce, Rockford, Illinois, USA) and imaged using an ECL system (Fusion FX7, Vilber Lourmat, Torcy, France). The band intensity was determined using Quantity One Software (Bio-Rad, Hercules, CA, USA).

### 2.5. Immunohistochemical Staining

After the colon tissues were paraffin-embedded and sectioned, immunohistochemical analyses were performed according to the method described. Briefly, goat polyclonal anti-human JAK2 and IL-23R antibodies were used as the primary antibody. Corresponding areas of sections were marked and high-power fields were counted at 200x magnification.

### 2.6. Statistical Analysis

Due to the individuals highly skewed distributions, comparison of values for all dates between IBD subgroups and control was performed by Mann-Whitney *U* test (2 groups compared) or Kruskal-Wallis test (more than 2 groups compared). All data were analyzed with Statistical Product and Service Solutions Vision 17.0 (SPSS, Chicago, Illinois, USA). Results were considered statistically significant only if the *P* value was less than 0.05.

## 3. Results

### 3.1. Levels of IL-12p40 and TL1A mRNA Were Upregulated in the Inflamed Intestinal Mucosa of UC Patients

Previously, we found that IL-12B and TNFSF15 genes polymorphisms were associated with UC in Chinese patients, which clearly showed that these two genes were involved in human susceptibility to UC.

**Figure 1:**
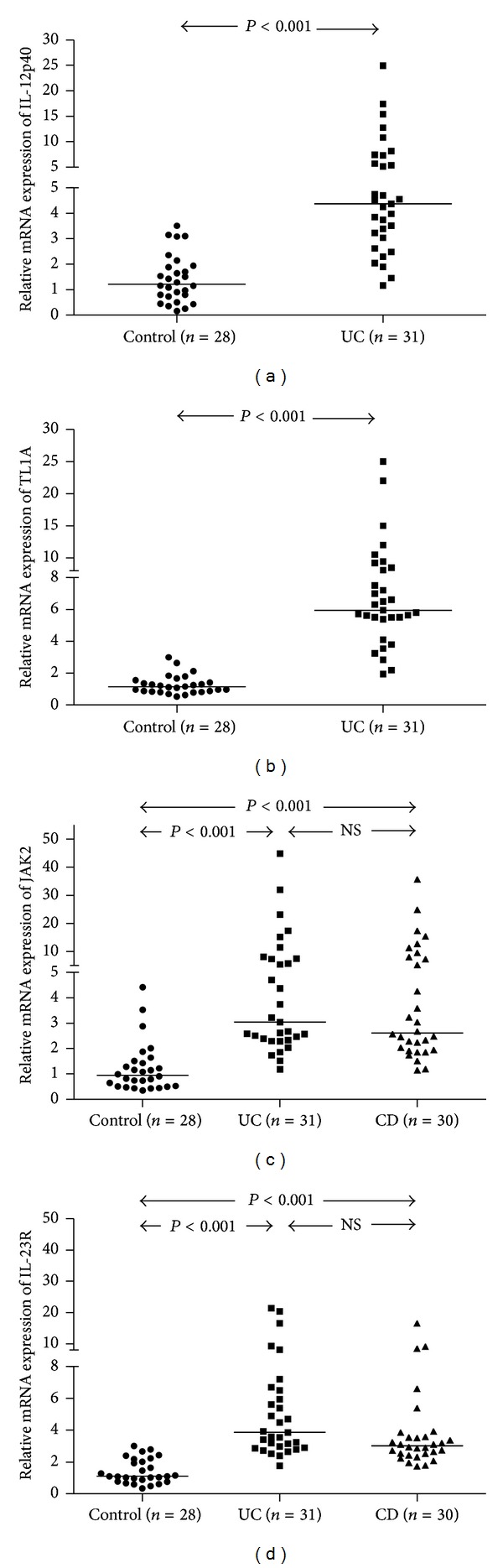
The mRNA expression of IL-23/Th17 pathway related genes in IBD patients. (a) and (b) The levels of IL-12p40 and TL1A mRNA expression were higher in UC patients than healthy controls (*P* < 0.001). (c) and (d) There was a highly significant increase in the expression of JAK2 and IL-23R in patients with UC and CD as compared to healthy controls (*P* < 0.001, for both comparisons), but no significant difference was observed between UC and CD patients. The level of mRNA expression was normalized to that of *β*-actin mRNA expression. Horizontal lines represent median values for each group and NS indicate no significance.

To further understand their biological functions, here we compared the relative IL-12p40 and TL1A mRNA expression between samples obtained at endoscopy from the UC patients and healthy controls. The relative level of IL-12p40 transcripts in colonic mucosa was significantly different between UC and healthy controls (mean ± SD: 6.01 ± 5.25 versus 1.43 ± 0.94, resp.; *P* < 0.001; see Figure 1(a)). In comparison to IL-12p40, we also observed a clear difference between UC and healthy controls regarding TL1A expression (UC: 7.52 ± 5.12, healthy controls: 1.27 ± 0.58; *P* < 0.001; see Figure 1(b)).

### 3.2. Serum IL-12B and TL1A Levels Were Increased in Patients with UC

**Figure 2:**
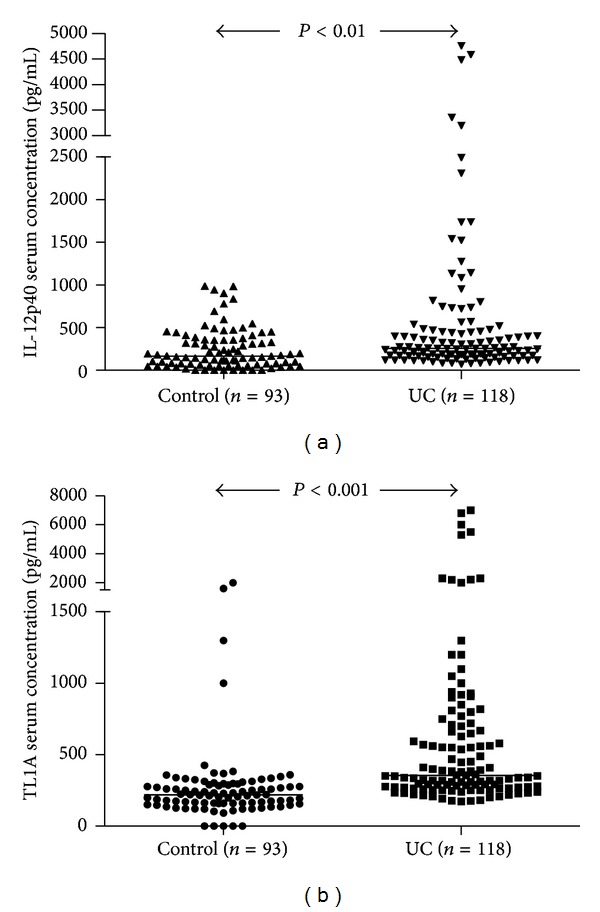
Serum IL-12p40 and TL1A levels were elevated in patients with UC. (a) Levels of IL-12p40 were higher in patients with UC (median, 95% CI: 255.59, 68.16–4756.60 pg/mL) than healthy controls (164.43, 0.1–986.43 pg/mL, *P* < 0.01). (b) Serum TL1A concentrations were significantly increased in UC (354.24, 172.78–7113.24 pg/mL) as compared with healthy controls (218.95, 0–2032.06 pg/mL, *P* < 0.001). *P* values refer to Mann-Whitney *U* test (for 2-group comparison). Horizontal lines represent median values for each group.

Since our tissue data clearly showed the local increase of IL-12p40 and TL1A, we then compared their serum concentrations in UC individuals and the healthy controls in the systemic levels. We found that the serum IL-12p40 levels were elevated in UC patients than that in healthy controls (255.59, 68.16–4756.60 versus 164.43, 0.1–986.43 pg/mL; *P* < 0.01; median, 95% CI; see Figure 2(a)) and serum TL1A concentrations were also increased in UC patients (354.24, 172.78–7113.24 pg/mL) compared with healthy controls (218.95, 0–2032.06 pg/mL, *P* < 0.001; see Figure 2(b)).

**Figure 3:**
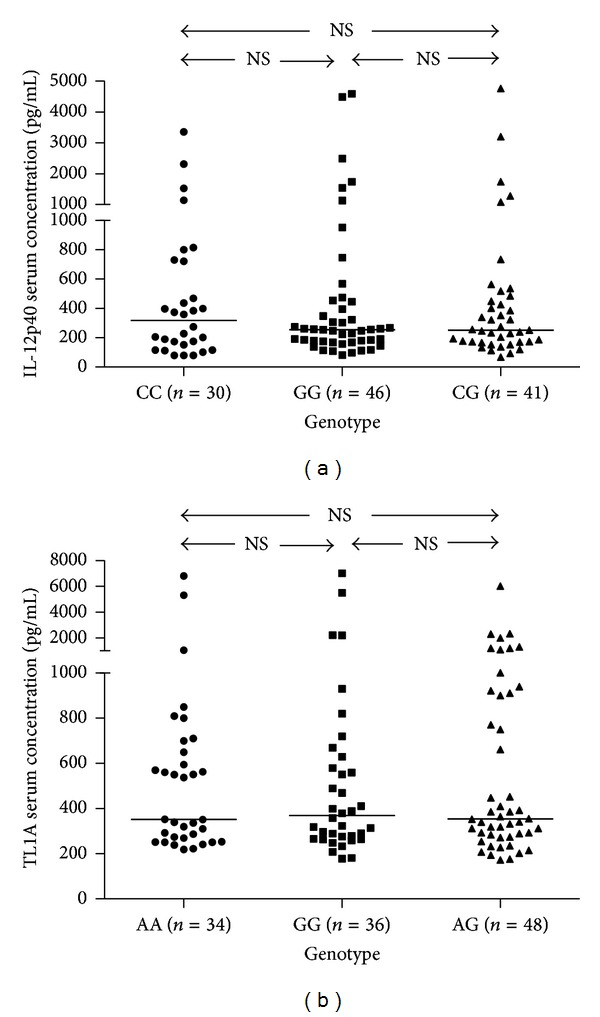
The relationship of polymorphism with serum IL-12p40 and TL1A levels in UC patients. (a) and (b) Among different genotype groups, no association was observed between the polymorphism and serum levels of IL-12p40 or TL1A in patients with UC (*P* > 0.05, for both comparisons). *P* values refer to Mann-Whitney *U* test (for 2-group comparison) and Kruskal-Wallis test (more than 2 groups compared). Horizontal lines represent median values for each group and NS indicate no significance.

To our knowledge, previous studies suggested that genotype-phenotype association played an important role in IBD, so we suspected that, whether the increase in serum IL-12p40 and TL1A levels was due to the genotype-phenotype association, the serum concentrations should be varying in different genotype. We investigated the effect of the distribution of rs6887695 or rs4263839 SNP on the serum levels of IL-12p40 and TL1A in UC patients, respectively. There were 71 of 118 UC patients genotyped allele C and 87 of 118 UC patients genotyped allele G for rs6887695, while 82 of 118 UC patients were genotyped allele A and 84 of 118 UC patients were genotyped allele G for rs4263839 (Figure 3). Results showed that there was no statistical difference among three genotype groups (*P* > 0.05; see Figure 3). Thus, the differences in IL-12p40 and TL1A expression are not related to their genotype.

### 3.3. Association of Serum IL-12p40 and TL1A Levels with Clinical Features of Patients with UC

We then examined whether the concentrations of IL-12p40 and TL1A were associated with clinical features of patients with UC. We analyzed serum IL-12p40 and TL1A levels in patients with UC after stratification by gender, age, course of disease, smoke habit, family history, extraintestinal manifestations, location, behavior, and treatments of disease (Table 3). Our results showed that the disease characteristics revealed no association on serum IL-12p40 or TL1A (*P* > 0.05), including the gender, age, smoke habit, and family history. Serum TL1A levels were higher in UC patients without extraintestinal manifestations (*P* = 0.032), which exhibited no statistically significant difference in serum IL-12p40 levels.

Among UC patients, serum IL-12p40 and TL1A levels were influenced by disease course, exhibiting higher values in UC patients with disease course less than 1.25 years (IL-12p40, *P* = 0.011; TL1A, *P* = 0.023). Comparisons among serum IL-12p40 and TL1A levels of UC patients with different disease behaviors exhibited significant differences (IL-12p40, *P* < 0.001; TL1A, *P* = 0.001), which were elevated in patients with initial onset than those with chronic relapse or chronic continuous IBD. Moreover, serum IL-12p40 and TL1A levels were higher in active disease compared with inactive disease (IL-12p40, *P* = 0.006; TL1A, *P* = 0.024). No association was established between disease location, treatments, and serum IL-12p40 or TL1A levels.

### 3.4. JAK2 and IL-23R mRNA Were Highly Expressed in IBD Patients

As for mRNA, different levels of JAK2 were observed in UC, CD, and healthy controls (5.63 ± 4.96 versus 5.75 ± 5.77 versus 1.23 ± 0.97, resp.; *P* < 0.001; see Figure 1(c)). Meanwhile, the relative average IL-23R mRNA expression was 5.76 ± 4.94 in UC patients and 3.90 ± 2.97 in CD patients, which were significantly elevated compared with healthy controls (1.41 ± 0.77; *P* < 0.001; see Figure 1(d)).

### 3.5. The Protein Expression of JAK2 and IL-23R Was Significantly Elevated in IBD Patients

**Figure 4:**
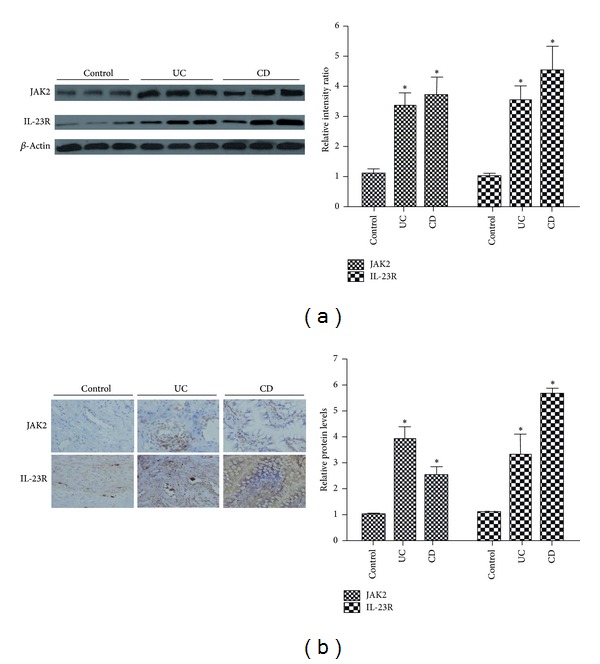
The expression levels of JAK2 and IL-23R protein in UC and CD patients. Compared with healthy controls, the local protein expression of JAK2 and IL-23R was enhanced in patients with UC and CD. (a) Quantitative analysis of the expressions of JAK2 and IL-23R relative to the intensity of *β*-actin by western blot. Autoradiograms were quantified with scanning densitometry. (b) Immunohisto chemical analysis for JAK2 and IL-23R quantification. (magnification: ×100). “∗”indicated *P* < 0.05 versus control group.

To confirm the expression of JAK2 and IL-23R, western blot and immunohistochemical analyses were performed on intestinal mucosal biopsies of 31 UC, 30 CD, and 28 healthy controls using primary antibodies that recognize the bioactive forms of JAK2 and IL-23R. The significant increase of JAK2 and IL-23R was found in UC and CD compared with healthy controls in colon tissues (Figure 4).

## 4. Discussion

Our study identified the abnormal IL-23/Th17 pathway expression levels both locally and systemically in IBD patients. The results indicated that UC patients had higher mRNA and serum levels of IL-12B and TL1A, while JAK2 and IL-23R levels were higher in UC and CD patients. Moreover, genotype-phenotype analysis showed that there was no significant correlation between the serum levels and different genotype groups of IL-12B or TL1A in UC patients. Nonetheless, some association was found between serum IL-12p40 and TL1A with the extraintestinal manifestations, disease course, and behavior of UC in our study.

Recent studies suggested that the genetic polymorphisms were associated with gene expressions and functions in IBD, which indicated that genetic variants could regulate individual's susceptibility to IBD [22–25]. Our unpublished data found that IL-12B, IL-23R, JAK2, and TNFSF15 genes polymorphisms were strongly associated with IBD in Chinese population. In this study we first demonstrated that their levels were elevated in IBD and then investigated whether genetic polymorphism could affect the expression of IL-23/Th17 pathway genes which led to different clinical features in Chinese IBD patients.

Since we found that IL-12B polymorphisms were involved in human susceptibility to UC in Chinese population, we observed a remarkable increase in the mRNA and protein level of IL-12p40 in mucosal biopsies and serum of UC patients. Currently, there was limited data about the phenotypic effects of IL-12B in Chinese population. However, recent studies suggested that IL-12p40 homodimer may have higher affinity for the IL-23 receptor than IL-12 receptor, and IL-12p40 delivery ameliorated dextran sulfate sodium-induced colitis by suppressing IL-17A production and inflammation in the intestinal mucosa [26]. Moreover, the secreted IL-12p40 was able to inhibit IL-23-mediated immune responses [22]. Therefore, it showed that IL-12p40 could be another novel target.

Our unpublished results found that the SNP rs6887695 of IL-12B was associated with UC pathogenesis. From a genotype-phenotype interaction standpoint, our results showed that the rs6887695 SNP polymorphism might be unable to fully affect the IL-12p40 serum levels, which suggested that the genetic polymorphisms located within these genes did not thoroughly explain the variations in disease phenotypes or IBD pathogenesis. Furthermore, we evaluated the possible relationship between serum IL-12p40 levels and clinical features of UC patients. Our results suggested that the elevation of serum IL-12p40 levels was only affected by disease course and behavior which suggested that the active UC patients with shorter course or initial onset had higher expression of serum IL-12p40. The similar results were also observed in the onset phase of autoimmune arthritis [27, 28]. Our results suggested that IL-12p40 may act as a molecular marker to indicate active disease on the early onset of UC patients, and therapy against IL-12p40 may delay the onset and reduce the UC severity.

TL1A, which represented a ligand for the death domain receptor 3 (DR3), was involved in clinical and experimental intestinal inflammation [16, 29]. TL1A induced Th17 immune response in cooperation with IL-23 in the CD pathogenesis [30]. Recent studies reported that the soluble TL1A levels increased in UC [31, 32]. Herein, we corroborated these findings by showing the increase of soluble TL1A levels in UC as well as the mucosal TL1A mRNA expression. It was the first evidence that the TL1A mRNA and protein expression levels were increased in Chinese UC patients.

Our results implied that the rs4263839 SNP polymorphism of TNFSF15, which was associated with UC patients in our previous data, might be unable to fully affect the serum TL1A levels. Then we evaluated the possible relationship of serum TL1A levels with clinical features of UC patients. Like IL-12p40, there was a significant correlation between serum TL1A levels and course or disease behavior. The polymorphism of decoy receptor 3 (DcR3), another TL1A receptor, was also associated with the pediatric onset UC [33]. Our results also suggested that serum TL1A level may act as a risk factor on the onset phase of active UC patients. Different from IL-12p40, we found that serum TL1A levels were higher in UC patients without extraintestinal manifestations, presumably because the patients in the cohort was limited and/or serum TL1A levels may be affected by the UC patient distribution.

As we know, IL-23/IL-23R activated JAK2/STAT3 to induce Th17 cell differentiation and this pathway contributed to the IBD pathogenesis [8–10]. Various studies delineated the effect of JAK2 on mediating intestinal inflammation [14, 34, 35]. Our unpublished results found that polymorphisms of IL-23R and JAK2 were associated with UC and CD, so we further demonstrated that the mRNA and protein levels of IL-23R and JAK2 were increased in intestine mucosa of IBD patients. Similarly, it was also reported that IL-23R-positive cells were significantly increased in NK cells and some kinds of T cells in IBD patients while the JAK2 mRNA expression was upregulated in CD patients [36, 37].

Finally, besides IL-12p40, TL1A, IL-23R, and JAK2 mentioned in this research, IL-23/Th17 pathway cytokines in IBD also included IL-23p19, STAT3, IL-17, and IL-22 [2, 3]. Our unpublished study did not find their polymorphisms to be associated with IBD in Chinese population. However, it was reported that Th17 cells may be responsible for the deleterious effects through IL-17 expression and/or the beneficial effects through IL-22 production, which meant that these cytokines may play potential roles in IBD [38, 39]. Moreover, IL-17 could activate stromal, endothelial, and epithelial cells to produce cytokines and chemokines, which not only help neutrophil recruit into tissues, but also induce inflammatory cytokine production by macrophages [40]. Thus, further investigations on the mechanisms of IL-23/Th17 cytokines are still needed.

## 5. Conclusion

Taken together, our data was the first comprehensive finding to evaluate the relationship between IL-23/Th17 pathway and IBD in Chinese population. We found that the expression of IL-23/Th17 pathway genes was both locally and systemically elevated in IBD patients. Furthermore, we demonstrated that the serum IL-12p40 and TL1A levels were not associated with the gene polymorphism but were highly expressed in the early onset and active condition of UC patients. Together, IL-23/Th17 pathway genes may represent important biomarkers of active inflammation in Chinese IBD patients, and they were new potential therapeutic targets for IBD.

## Figures and Tables

**Table 1:** Demographic characteristics and clinical features of the patients with UC, CD, and healthy controls.

Characteristics		UC (*n* = 118 (%))	CD (*n* = 30 (%))	Healthy controls (*n* = 93 (%))
Male		54 (45.76)	19 (63.33)	65 (69.89)
Age (years) mean ± SD (range)		40.19 ± 13.84 (10–67)	33.80 ± 11.11 (17–61)	39.83 ± 13.07 (21–88)
Course of disease (years) Mean ± SD (range)		2.45 ± 3.26 (1.25, 0.003–23)	3.16 ± 4.02 (1.29, 0.08–16)	—
Smoke habit		24 (20.34)	7 (23.33)	—
Family history		13 (11.02)	0	—
Extraintestinal manifestations		33 (27.97)	16 (53.33)	—

Disease location				
UC	CD			
Left sided colitis	Colon	35 (29.66)	7 (23.33)	—
Extensive colitis	Ileocolon	36 (30.51)	9 (30.00)	—
Proctitis	Ileum	47 (39.83)	14 (46.66)	—

Disease behavior				
UC	CD			
Initial onset	Stricturing	54 (45.76)	4 (13.33)	—
Chronic relapse	Nonstricturing, Nonpenetrating	57 (48.31)	19 (63.33)	—
Chronic continuous	Penetrating	7 (5.93)	7 (23.33)	—
Active disease		90 (76.27)	20 (66.67)	—
Inactive disease		28 (23.73)	10 (33.33)	—

Treatment				
5-ASA/SASP		103 (87.29)	23 (76.67)	—
Steroid		52 (44.07)	9 (30.00)	—
Antibiotics		28 (23.73)	2 (6.67)	—
Immunosuppressive		9 (7.63)	5 (16.67)	—
Infliximab		1 (0.85)	4 (13.33)	—
Operation		0	5 (16.67)	—

SD: standard deviation; 5-ASA: 5-aminosalicylate; SASP: sulfasalazine.

**Table 2:** Primer sets used for quantitative real-time PCR.

Gene	Primer sets	Length (bp)
IL-12B		
Forward	GACAAGTAGTTATGGCTAAGGACATGA	102
Reverse	AGGGATTCCAGATTTTCTTTGCA	
TNFSF15		
Forward	GAGGCCTGTGTGCAGTTCCA	182
Reverse	CCTAGTTCATGTTCCCAGTGCAGA	
JAK2		
Forward	GTGTTCCATTTGATAGAACTTTTGAAGA	105
Reverse	ATTATTGTTCCAGCATTCTGTCATGA	
IL-23R		
Forward	TGGGTCCAAGCAGCAAACGCAC	105
Reverse	CTCAGCCCTGGAAATGACGGCTG	
*β*-actin		
Forward	CTCCATCCTGGCCTCGCTGT	268
Reverse	GCTGTCACCTTCACCGTTCC	

**Table 3:** Serum IL-12p40 and TL1A levels in patients with ulcerative disease (UC) according to disease characteristics.

Characteristics	UC (*n* = 118)	IL-12p40 (pg/mL) Median (range)	TL1A (pg/mL) Median (range)
Gender			
Male	54	275.12 (82.54–4756.60)	353.17 (178.15–5989.46)
Female	64	252.84 (68.16–4581.92)	355.85 (172.78–7113.23)
Age (years)			
≤40	60	251.21 (68.16–4482.94)	339.75 (172.78–5425.89)
>40	58	256.91 (79.33–4756.60)	386.99 (182.45–7113.23)
Course of disease (years)			
≤1.25	62	306.21 (79.33–4756.60)*	433.65 (172.78–7113.23)*
>1.25	56	214.40 (68.16–3355.24)	319.35 (176.00–5425.89)
Smoke habit			
Yes	24	291.60 (82.54–4756.60)	385.38 (218.95–5989.46)
No	94	252.84 (68.16–4581.92)	353.17 (172.78–7113.23)
Family history			
Yes	13	246.33 (79.33–1131.80)	318.81 (207.14–5425.89)
No	105	257.01 (68.16–4756.60)	358.54 (172.78–7113.23)
Extraintestinal manifestations			
Yes	33	229.34 (79.33–3355.24)	310.22 (172.78–2198.54)*
No	85	258.43 (68.16–4756.60)	388.60 (176.00–7113.23)
Active disease	90	290.04 (68.16–4756.60)**	390.75 (172.78–7113.23)*
Inactive disease	28	185.60 (80.91–3193.59)	293.04 (176.00–2305.60)
Disease location			
Extensive colitis	36	280.63 (82.54–4756.60)	347.26 (172.78–6831.63)
Distal colitis	82	252.84 (68.16–4581.92)	361.22 (176.00–7113.23)
Disease behavior			
Initial onset	54	361.95 (79.33–4756.60)**	545.21 (207.14–7113.23)**
Chronic relapse/chronic continuous	64	214.39 (68.16–3355.24)	312.90 (172.78–5425.89)
Treatment			
SASP/5-ASA monotherapy	66	239.69 (79.33–4581.92)	359.51 (176.00–6831.63)
Other drugs	52	268.61 (68.16–4756.60)	353.17 (172.78–7113.23)

*P < 0.05, **P < 0.01.
